# Efficacy of vitamin C and ethanolic extract of *Sesamum indicum* in promoting fertility in male Wistar rats

**DOI:** 10.4103/0974-1208.63115

**Published:** 2010

**Authors:** EA Ashamu, EO Salawu, OO Oyewo, AW Alhassan, OA Alamu, AA Adegoke

**Affiliations:** 1 Department of Anatomy, Faculty of Basic Medical Sciences, College of Medicine, Ladoke Akintola University of Technology, Ogbomoso, Nigeria; 2 Department of Physiology, Faculty of Basic Medical Sciences, College of Medicine, Ladoke Akintola University of Technology, Ogbomoso, Nigeria; 3 Department of Human Physiology, Faculty of Medicine, Ahmadu Bello University, Zaria, Nigeria

**Keywords:** Antioxidant, fertility, *Sesamum indicum*, sperm count, vitamin C

## Abstract

**AIMS:**

This study investigates the efficacy of ethanolic extract of *Sesamum indicum* (EES), vitamin C (VC), and EES + VC in promoting fertility and finding a possible link between their profertility effects and their antioxidant activities.

**MATERIALS AND METHODS:**

Forty adult male Wistar rats [Body weight (BW) 186.56 ± 0.465 g] were randomly analyzed into four groups of ten rats each: Control, EES_G_ (EES only), VC_G_ (vitamin C only), and EES + VC_G_ (EES in conjunction with vitamin C). Control was given 5 ml/kg BW/day of normal saline orally; EES_G_ was administered 0.3 g/kg BW/day of EES; VC_G_ was administered 15 mg/kg BW/ day of VC; while EES + VC_G_ was administered both 0.3 g/kg BW/day of EES and 15 mg/kg BW/day of VC. All treatments were for 10 weeks.

**STATISTICAL ANALYSIS USED:**

Independent-sample T test was used to analyze the obtained results.

**RESULTS:**

The results obtained showed that EES, VC, and more importantly EES + VC are capable of significantly increasing BW gain, seminal parameters, testosterone level, and body antioxidant activities.

**CONCLUSIONS:**

These findings lead to the conclusion that EES + VC as well as ESS and VC promote fertility due to both their testosterone-increasing effects and their antioxidant effects.

## INTRODUCTION

Because infertility are highly diversified in etiology, their treatments also require diversified approach. Some orthodox medications and traditional medications have proved really useful in the treatment of infertility.[1] However, orthodox medications often have the disadvantage of strong side effects and too high costs.[2]

Some traditional doctors claim to use ethanolic extract of *Sesamum indicum* (EES) and citrus fruits [sources of vitamin C (VC)] to promote fertility. If we could establish that EES in conjunction with VC significantly promotes fertility, then there is a possibility that some cases of infertility could be treated cheaply with EES and VC. Many studies have also shown that antioxidants can enhance fertility either directly or indirectly and that most plants rich in antioxidants have the tendency to increase sperm counts, motility, and enhance sperm morphology.[3,4] *Sesamum indicum* has lots of components appropriate for detoxification and quite a number of antioxidants such as sesamol, sesamolin sesamin, butylated hydroxytoluene, sesaminol triglucoside, and sesaminol diglucoside.[5] Similarly VC has been shown to be a free radical scavenger and to protect against lipid peroxidation. This ability has been reported to increase peripheral testosterone level and also attenuate testicular toxicity.[6] There is, therefore, a high possibility that EES acting alone, or with VC, can promote fertility and/or treat infertility.

This study aims to investigate the efficacy of EES, VC, and EES + VC in promoting fertility, and find a possible link between their profertility effects and antioxidant activities.

## MATERIALS AND METHODS

Forty adult male Wistar rats of average body weight 186.56 ± 0.465 g were used for this study. They were inbred in the Animal House of the Faculty of Basic Medical Sciences, Ladoke Akintola University of Technology, Ogbomoso, Nigeria. The animals were transferred to the research section, and acclimatized over a period of 2 weeks. The research section was adequately ventilated and maintained at a temperature of 24-26°C, relative humidity of 70-75%, and at light/dark cycle of 12/12 hours. The rats were fed with commercially available rat pellet. The maintenance, care, and treatments of the rats comply with National Institutes of Health guidelines for the humane use of laboratory animals, and those of Ladoke Akintola University of Technology, Ogbomoso, Nigeria.

The VC used was manufactured by, and obtained from, Tuyil Pharmaceutical Company, Ilorin, Nigeria. *Sesamum indicum* was obtained from Forest Research Institute of Nigeria (FRIN), Ibadan, Nigeria. The *Sesamum indicum* was authenticated in the Herbarium Unit of the Department of Pure and Applied Biology, Ladoke Akintola University of Technology, Ogbomoso, Nigeria, and assigned Specimen number S11387.

EES was prepared following the exact method described by Kizhiyedathu *et al*.[5]

### Grouping of animals and treatment

The rats [average body weight (BW) 186.56 ± 0.465 g] were randomly analyzed into four groups of 10 rats each: Control, EES_G_ (extract of *Sesamum indicum* only), VC_G_ (vitamin C only), and EES + VC_G_ (EES indicum in conjunction with VC). Control was given 5 ml/kg BW of normal saline orally per day; EES_G_ was administered 0.3 g/kg BW of EES per day; VC_G_ was administered 15 mg/kg BW of VC per day; while EES + VC_G_ was administered both 0.3 g/kg BW of EES and 15 mg/kg BW of VC per day. All treatments were for 10 weeks.

### Animal sacrifice and collection of samples

Twenty-four hours after the last treatment, each animal was weighed and then sacrificed by cervical dislocation. Up to 4.0 ml blood samples were collected via cardiac puncture. Blood sample obtained from each rat was divided into two portions: 2.0 ml in a plain bottle and the other 2.0 ml in an ethylenediaminetetraacetic acid bottle. Plasma and serum were obtained by centrifugation at 3000 rpm for 20 minutes. Testis and caudal epididymis were excised from each rat.

### Collection of data and statistical analysis

The left testis of each rat was homogenized (using soft tissue homogenizer manufactured by Omni International, USA) for tissue superoxide dismutase (SOD) and catalase (CAT) activities, and malondialdehyde (MDA) concentration. Sperm count, motility, morphology, and life-death ratio of the rats' spermatozoa were carried out from the epididymis. Serum testosterone level was determined. Plasma and tissue SOD and CAT activities, and MDA concentrations were determined using the methods described by Fridovich,[7] Sinha,[8] and Varshney and Kale,[9] respectively.

The data obtained are presented as mean ± SD. The control and test groups were compared using Independent-sample T test. Significance level was taken at *P*-value <0.05.

## RESULTS

### Body weight and testicular weight

Comparing their final and initial weights showed that there was significant body weight gain in all the groups over the period of the experiment. The weight gain in EES_G_ group as well as EES + VC_G_ was, however, significantly (*P*-value <0.01) higher than that of the control, while weight increase in VC_G_ showed no significant difference from that of the control [Table 1].

**Table 1:** Body weight and testicular weight

	Control	EES_G_	VC_G_	EES + VC_G_
Weight before sacrifice (g)	202.6 ± 0.290	211.5 ± 0.365	205.3 ± 0.329	212.6 ± 0.346
Initial weight (g)	185.9 ± 0.257	186.1 ± 0.291	187.6 ± 0.299	186.3 ± 0.333
Weight increase (g)	16.7 ± 0.238	25.4 ± 0.289**	17.7 ± 0.252	26.3 ± 0.311**
Testicular weight (g)	0.660 ± 0.033	0.714 ± 0.029*	0.624 ± 0.024	0.746 ± 0.032**

* *P*-value < 0.05

** *P*-value < 0.01

The testicular weight of VC_G_ showed no significant (*P*-value >0.05) difference from that of the control, while the testicular weight of EES_G_ (*P*-value <0.05) and EES + VC_G_ (*P*-value <0.01) was found to be significantly higher than that of the control [Table 1].

### Sperm count (SC), life-death ratio (LDR) of sperm cells, sperm motility (SM), sperm morphology (SMP), and serum testosterone level (STL)

SC for EES_G_ and EES + VC_G_ treated animals was significantly (*P*-value <0.01) higher than that of the control, whereas SC for VC_G_ alone showed no significant difference (*P*-value >0.05) from that of the control. Likewise, LDR of EES_G_ and EES + VC_G_ was found to be significantly (*P*-value <0.01) higher than that of the control, while VC showed no significant (*P*-value >0.05) difference from the control [Table 2].

**Table 2:** Sperm count, life-death ratio of sperm cells, sperm motility, sperm morphology, and serum testosterone level

	Control	EES_G_	VC_G_	EES + VC_G_
Sperm count (million cells/mm^3^)	98.1 ± 0.247	119.7 ± 0.296**	105.3 ± 0.349	129.5 ± 0.290**
Life-death ratio of sperm cells (%)	4.03 ± 0.081	4.89 ± 0.077**	4.18 ± 0.070	5.16 ± 0.083**
Sperm motility (%)	79.5 ± 0.333	89.6 ± 0.244*	84.0 ± 0.295	91.0 ± 0.280**
Sperm morphology (%)	78.2 ± 0.303	81.5 ± 0.292	76.5 ± 0.241	84.5 ± 0.299
Serum testosterone level (ng/ml)	21.34 ± 0.212	24.9 ± 0.195*	24.8 ± 0.158*	25.1 ± 0.142*

* *P* > 0.05

** *P* < 0.01

EES_G_ and EES + VC_G_ groups had SM that was significantly (*P*-value <0.05 and <0.01, respectively) higher than that of the control, whereas SM for VC_G_ was not significantly (*P*-value >0.05) different from that of the control. SMP was, however, not significantly (*P*-value >0.01) different in all the test groups compared with the control [Table 2].

There was significant (*P*-value <0.05) increase in STL in all the test groups compared to the control [Table 2].

### Plasma and testicular superoxide dismutase activity

Plasma and testicular SOD activity were found to be significantly higher in all the treated groups (*P*-value <0.01 in EES_G_ and EES + VC_G_; and *P*-value <0.05 in VC_G_) compared to the control [Table 3].

**Table 3:** Plasma and testicular superoxide dismutase activity

	Control	EES_G_	VC_G_	EES + VC_G_
Plasma SOD activity	1.643 ± 0.040	1.837 ± 0.037**	1.762 ± 0.031*	1.884 ± 0.043**
Testicular SOD activity	1.602 ± 0.048	1.815 ± 0.033**	1.781 ± 0.045*	1.896 ± 0.051**

* *P* < 0.05

** *P* < 0.01

### Plasma and testicular catalase activity

While testicular catalase (CAT) activity was found to be significantly higher in all the treated groups (*P*-value <0.05) compared to the control, plasma CAT activity was only significantly (*P*-value <0.05) higher in EES + VC_G_ [Table 4].

**Table 4:** Plasma and testicular catalase activity across the groups

	Control	EES_G_	VC_G_	EES + VC_G_
Plasma CAT activity	0.484 ± 0.045	0.568 ± 0.039	0.576 ± 0.043	0.670 ± 0.037*
Testicular CAT activity	0.456 ± 0.040	0.607 ± 0.033*	0.604 ± 0.033*	0.671 ± 0.050*

* *P* < 0.05

### Plasma and testicular malondialdehyde concentration

Plasma Malondialdehyde (MDA) concentration was found to be significantly lower in all the treated groups (*P*-value <0.05 in EES_G_ and VC_G_; and *P*-value <0.01 in EES + VC_G_) compared to the control. In a similar way, testicular MDA concentration was found to be significantly lower in all the treated groups (*P*-value <0.05 in EES_G_; and *P*-value <0.01 in VC_G_ and EES + VC_G_) compared to the control [Table 5].

**Table 5:** Plasma and testicular malondialdehyde concentration across the groups

	Control	EES_G_	VC_G_	EES + VC_G_
Plasma MDA concentration (μg/g protein)	1359.9 ± 1.202	1160.0 ± 1.428*	1181.1 ± 1.387*	1042.1 ± 1.34**
Testicular MDA concentration (μg/g protein)	1320.6 ± 0.861	1170.5 ± 1.474*	1170.0 ± 1.28**	1010.0 ± 1.53**

* *P* < 0.05

** *P* < 0.01

## DISCUSSION

Even though VC is very important for normal health condition by its antioxidant and detoxification actions,[6] VC all by itself does not significantly affect the increase in body weight (weight gain). This explains the nonsignificant increase in weight gain of VC_G_ compared to the control [Table 1]. This is in support of the findings of Ping-Chi *et al*.[10] On the contrary, EES acting alone (EES_G_) and in conjunction with VC (EES + VC_G_) significantly increases weight gain. The significant increase in weight gain in EES_G_ can be linked to the high fat composition[11] and its high caloric content. Similar findings have earlier being documented by Salawu *et al*.[12] that methanolic extract of sesame has the ability to annul the weight reducing effect of hexane extract of Jatropha curcus seed oil; Ukwenya *et al*.[13] that *Sesamum indicum* significantly improved weight gain; and by Hanefy *et al*.[14] that methanolic extract of sesame (MES) prevents protein energy malnutrition, thus improving the growth performance in animals. However, the significantly higher weight gain noticed in EES + VC_G_ reveals the possibility that EES in conjunction with VC have a synergistic effect on body weight, after all VC acting alone does not significantly affect body weight [Table 1]. A completely similar trend to that of body weight was noticed for absolute testicular weight throughout the groups.

The observed ability of VC to increase SC [Table 2] is in line with the outcomes of the study by Comhaire *et al*.[15] that antioxidants increase sperm concentration, and goes along with Sikka *et al*.[16] that a balance of the benefits and risks from ROS and antioxidants appears to be necessary for the survival and functioning of spermatozoa. This spermatogenic effect of VC can easily be linked to its antioxidant effects, which have been scientifically proved to be cytoprotective, and to attenuate testicular toxicity. They preserve the existing, and even make available more active/functional, semineferous tubules with good cyto-architecture. In a similar way, but with more effect, EES significantly increased SC [Table 2]. Salawu *et al*.[12] earlier documented a similar finding on methanolic extract of sesame. The SC-increasing effect of EES + VC was more marked and seems to be additive rather than synergistic as in the case of BW. This finding is able to provide some basis for the use of sesame in addition to citrus fruits for profertility effects and for preventing infertility by the traditional doctors/in folklore medicine.

The life-death ratio (LDR) of the EES + VC_G_ was far higher than the LDR of all other groups [Table 2]. This further establishes and tends to establish the profertility effect of oral administration of EES in conjunction with VC. This outcome is justifiable by the claims in the review by Sikka *et al*.[16] that antioxidants are necessary for the survival of spermatozoa, and by the claims of folklore medicine.

The observed significant increase in SM and SMP in ESS + VC_G_ [Table 2] further establishes the ability of EES acting in conjunction with VC to potentiate more efficient spermatogenesis and fertility. While VC acting alone did not significantly affect both SM and SMP as against the findings of Ping-Chi *et al*.[10] that VC protects spermatozoa from the loss of motility, EES all alone significantly increased both the SM and SMP, which is in support of the findings of Shittu *et al*.[17] The increment in SM and SMP in EES_G_ was, however, lower than that of EES + VC_G_ [Table 2]. This justifies the combination of EES and VC in traditional medicine for fertility issues, since “effect of EES + VC acting together” greater than “effect of VC and/or effect of EES acting separately.”

The significant increase in both plasma and testicular SOD activity [Table 3] and CAT activity [Table 4] in all the treated groups is in support of the antioxidant activities of vitamin C[6] and sesame.[5] These findings are further reinforced by the significantly reduced plasma and testicular MDA contraction. The MDA concentration is lowered in all the treated groups because of their increased SOD and CAT activities.

VC acting alone, EES acting alone, and VC + EES acting together significantly increased blood testosterone level. This finding is parallel to the observations of Salawu *et al*.,[12] Ukwenya *et al*.,[13] and Shittu *et al*.[17] that sesame significantly increases blood testosterone level. This finding shows that EES and VC whether acting singly or together affect the hypothalamo-pituitary-testicular axis, thus elevating blood testosterone level. Therefore, the profertility effects of EES and VC are linked to their ability to raise the blood testosterone level, and not only to their antioxidant activities. In other words, it is scientifically reasonable to attribute the profertility effects of EES and VC to both their antioxidant effects [6 and 11, respectively] [Tables 3–5] and their testosterone level increasing effect [12 and 6, respectively] [Table 2].
